# Alcohol, Intraocular Pressure, and Open-Angle Glaucoma: A Systematic Review and Meta-analysis

**DOI:** 10.1016/j.ophtha.2022.01.023

**Published:** 2022-06

**Authors:** Kelsey V. Stuart, Kian Madjedi, Robert N. Luben, Sharon Y.L. Chua, Alasdair N. Warwick, Mark Chia, Louis R. Pasquale, Janey L. Wiggs, Jae H. Kang, Pirro G. Hysi, Jessica H. Tran, Paul J. Foster, Anthony P. Khawaja, Modifiable Risk Factors for Glaucoma Collaboration

**Affiliations:** 1 NIHR Biomedical Research Centre, Moorfields Eye Hospital NHS Foundation Trust & UCL Institute of Ophthalmology, London, United Kingdom; 2 Department of Ophthalmology, University of Calgary, Alberta, Canada; 3 Department of Public Health and Primary Care, Institute of Public Health, University of Cambridge School of Clinical Medicine, Cambridge, United Kingdom; 4 UCL Institute of Cardiovascular Science, London, United Kingdom; 5 Department of Ophthalmology, Icahn School of Medicine at Mount Sinai, New York, New York; 6 Department of Ophthalmology, Massachusetts Eye and Ear, Harvard Medical School, Boston, Massachusetts; 7 Department of Medicine, Brigham and Women’s Hospital, Harvard Medical School, Boston, Massachusetts; 8 Department of Ophthalmology, King’s College London, St. Thomas’ Hospital, London, United Kingdom; 9 Department of Twin Research & Genetic Epidemiology, King’s College London, St. Thomas’ Hospital, London, United Kingdom

**Keywords:** Alcohol, Intraocular pressure, Meta-analysis, Open-angle glaucoma, Systematic review, ALDH2, aldehyde dehydrogenase 2, BWHS, Black Women’s Health Study, CI, confidence interval, GC-IPL, ganglion cell-inner plexiform layer, GRADE, Grading of Recommendations Assessment, Development and Evaluation, IOP, intraocular pressure, NHS/HPFS, Nurses’ Health Study and Health Professionals Follow-Up Study, OAG, open-angle glaucoma, OHT, ocular hypertension, OR, odds ratio, POAG, primary open-angle glaucoma, RNFL, retinal nerve fiber layer, ROBINS-E, Risk Of Bias In Non-randomized Studies of Exposures, RR, rate ratio

## Abstract

**Topic:**

This systematic review and meta-analysis summarizes the existing evidence for the association of alcohol use with intraocular pressure (IOP) and open-angle glaucoma (OAG).

**Clinical Relevance:**

Understanding and quantifying these associations may aid clinical guidelines or treatment strategies and shed light on disease pathogenesis. The role of alcohol, a modifiable factor, in determining IOP and OAG risk also may be of interest from an individual or public health perspective.

**Methods:**

The study protocol was preregistered in the Open Science Framework Registries (https://osf.io/z7yeg). Eligible articles (as of May 14, 2021) from 3 databases (PubMed, Embase, Scopus) were independently screened and quality assessed by 2 reviewers. All case-control, cross-sectional, and cohort studies reporting a quantitative effect estimate and 95% confidence interval (CI) for the association between alcohol use and either IOP or OAG were included. The evidence for the associations with both IOP and OAG was qualitatively summarized. Effect estimates for the association with OAG were pooled using random effects meta-analysis. Studies not meeting formal inclusion criteria for systematic review, but with pertinent results, were also appraised and discussed. Certainty of evidence was assessed using the Grading of Recommendations Assessment, Development and Evaluation (GRADE) framework.

**Results:**

Thirty-four studies were included in the systematic review. Evidence from 10 studies reporting an association with IOP suggests that habitual alcohol use is associated with higher IOP and prevalence of ocular hypertension (IOP > 21 mmHg), although absolute effect sizes were small. Eleven of 26 studies, comprising 173 058 participants, that tested for an association with OAG met inclusion criteria for meta-analysis. Pooled effect estimates indicated a positive association between any use of alcohol and OAG (1.18; 95% confidence interval [CI], 1.02–1.36; *P* = 0.03; *I*^*2*^ = 40.5%), with similar estimates for both prevalent and incident OAG. The overall GRADE certainty of evidence was very low.

**Conclusions:**

Although this meta-analysis suggests a harmful association between alcohol use and OAG, our results should be interpreted cautiously given the weakness and heterogeneity of the underlying evidence base, the small absolute effect size, and the borderline statistical significance. Nonetheless, these findings may be clinically relevant, and future research should focus on improving the quality of evidence.

Glaucoma comprises a heterogeneous group of diseases characterized by progressive optic neuropathy and visual field loss and is the leading cause of irreversible blindness worldwide.^1^^,^^2^ Global prevalence is estimated at 76 million and is projected to increase to 112 million by 2040.^1^ The precise pathogenesis of primary open-angle glaucoma (POAG), the most common form of the disease, is not fully understood, but the final disease pathway is marked by retinal ganglion cell apoptosis and optic nerve fiber loss.^2^ Prevailing hypotheses implicate intraocular pressure (IOP)-mediated mechanical stress, as well as various ocular vascular risk factors, as mediators of this process.^2^^,^^3^ It is likely that proximal determinants of POAG represent a complex interplay of genetic, environmental, anatomic, and physiologic factors.^2^ Currently, IOP remains the major modifiable risk factor for POAG, but there is considerable interest in identifying other potentially modifiable factors that may complement existing treatment strategies or shed light on disease pathogenesis.

Alcohol use is implicated in a multitude of chronic diseases across various organ systems and is the seventh leading cause of death and disability worldwide.^4^, ^5^, ^6^ The acute effects of alcohol on the human eye include a transient, seemingly dose-dependent reduction in IOP^7^, ^8^, ^9^, ^10^, ^11^, ^12^, ^13^, ^14^ and increase in blood flow to the optic nerve head,^13^^,^^15^ theoretically conferring a protective benefit against the development of glaucoma. Chronic alcohol use, however, is associated with a host of neurodegenerative, cardiovascular, and endocrine disorders, as well as systemic biochemical and physiologic derangements, and the long-term or indirect roles these may play in glaucoma are unclear.^4^^,^^5^

In contrast to the short-term ocular hypotensive effects of alcohol, a number of epidemiologic studies have reported cross-sectional associations between alcohol use and higher IOP or prevalence of ocular hypertension (OHT),^16^, ^17^, ^18^, ^19^, ^20^ but this is not always a consistent finding.^21^^,^^22^ There is also evidence to suggest that any association with IOP may be mediated by both sex and glaucoma status.^18^^,^^20^ Additionally, most observational studies exploring the association between alcohol use and glaucoma have yielded nonsignificant results, with both cross-sectional^16^^,^^23^, ^24^, ^25^, ^26^, ^27^, ^28^ and longitudinal studies^29^, ^30^, ^31^ failing to demonstrate a consistent association.

Existing reviews on the subject are limited to qualitative analyses within the context of broader review topics,^32^, ^33^, ^34^, ^35^, ^36^, ^37^ and, to the best of our knowledge, there have been no published systematic reviews and meta-analyses exploring the potential role that alcohol may play in determining IOP and glaucoma risk. Our research question, using the PECO (Population, Exposure, Comparator, Outcomes) framework, was in the general adult population (population), what is the effect of habitual alcohol consumption (exposure) on IOP and open-angle glaucoma (OAG) (outcomes) compared with those who do not consume alcohol (comparison)? A better understanding of these associations may offer insight into potential mechanisms of glaucomatous optic neuropathy, direct future research, and inform clinical advice or guidelines. It also may be of interest to individuals wanting to learn how modifiable lifestyle factors, such as alcohol consumption, may influence IOP or the risk for glaucoma.

## Methods

This study aimed to address the association between alcohol use with IOP and OAG in adults through systematic review and meta-analysis of observational studies. As such, it was conducted in accordance with the Meta-analysis of Observational Studies in Epidemiology guidelines.^38^ The study protocol was preregistered and published online in the Open Science Framework Registries (https://osf.io/z7yeg).^39^ Because this study involved only review and synthesis of existing literature, it was exempt from Institutional Review Board approval.

### Eligibility Criteria for Considering Studies for This Review

Alcohol use was defined as current or prior habitual consumption of any amount or type of alcohol. Open-angle glaucoma was chosen as an outcome measure because many studies do not differentiate between primary and secondary forms of OAG. Given that the potential exclusion of these studies may have limited our findings and that POAG constitutes the majority of OAG cases, this expanded definition was considered appropriate. We aimed to include all relevant case-control, cross-sectional, and cohort studies.

### Search Methods for Identifying Studies

One author (K.V.S.) systematically conducted a search of 3 databases (PubMed, Embase, and Scopus) to identify relevant articles published up to May 14, 2021, using the search strategies described in Appendix A (available at www.aaojournal.org). Independent review of retrieved titles and abstracts was conducted by 2 authors (K.V.S. and K.M.), and all articles deemed relevant to our research question were retrieved for full-text review. A manual search of the reference lists of all included studies and previous reviews was also performed by the same 2 authors. Any inconsistencies were resolved by consensus agreement or by consultation with a third reviewer (A.P.K.), when necessary.

### Study Selection

Full-text articles were required to meet the following inclusion criteria for the purposes of the systematic review: (1) reported alcohol use in keeping with our exposure definition; (2) reported IOP or OAG as the outcome measure; (3) reported the measure of association as an effect estimate with a 95% confidence interval (CI) or standard error, or allowed for the calculation of these measures from published raw data; and (4) study participants were 18 years of age or older. Studies were excluded if they were (1) reviews, letters, editorials, case reports, case series, conference abstracts, or animal studies; or (2) published in a non-English language. Articles not meeting formal criteria for systematic review but that were relevant to the study question were reviewed in full and pertinent findings reported in the “Discussion” section for context. When multiple publications from the same study population were available, we included the study that best addressed our research question. Preference was given to (1) studies with the correct exposure and outcome definitions, (2) prospective studies, (3) larger studies, and (4) studies with greater adjustment for confounding variables. This study selection process was performed independently by 2 authors (K.V.S. and K.M.) with arbitration by a third reviewer (A.P.K.) if necessary.

### Data Collection and Risk of Bias Assessment

For each included study, the following data were extracted using a standardized data collection tool: (1) first author name, (2) year of publication, (3) study name and country, (4) demographics of study participants, (5) study design, (6) number of study participants, (7) definition of alcohol exposure, (8) definition of IOP or OAG outcome, (9) effect estimate plus 95% CI or standard error, and (10) confounding variables adjusted for.

Studies were grouped according to their main outcome measure(s): (1) IOP (as either a continuous or categorical measure), (2) OAG (as either prevalent or incident cases). If studies addressed more than 1 outcome, these were reported separately.

A risk of bias assessment was independently performed by 2 authors (K.V.S. and K.M.), using a tool designed by the Grading of Recommendations Assessment, Development and Evaluation (GRADE) Working Group to assess the effects of environmental exposures on health outcomes.^40^ This tool is modeled on the established Risk Of Bias In Non-randomized Studies of Interventions instrument^41^ and was designed by the Risk Of Bias In Non-randomized Studies of Exposures (ROBINS-E) collaborative project to help guide the development of the final ROBINS-E instrument. Specific risk of bias domains assessed included confounding, selection of participants, classification of exposure, departures from intended exposure, missing data, measurement of outcomes, and selection of reported results. Inconsistencies were resolved in the manner described previously.

### Data Synthesis and Analysis

Because of considerable heterogeneity in the definition of both alcohol exposure and IOP across included studies, meta-analysis of this association was not deemed appropriate. Likewise, meta-analysis of the association between alcohol use and OAG was limited to the comparison of any alcohol use (exposure group) with no alcohol use (reference group). Studies reporting effect estimates for different levels or categories of alcohol exposure (e.g., former/current drinker, number of drinks per day/week, grams of alcohol consumed per day/week) were included, and strata-specific results were pooled using inverse variance-weighted, fixed-effects meta-analysis to obtain a single effect estimate for each study. This model was chosen because it was assumed that there would be no statistical, clinical, or methodological heterogeneity between effect estimates derived from a single study.

Studies were excluded from meta-analysis if they met any of the following criteria: (1) did not provide a multivariable-adjusted effect estimate or (2) the reference group was not comparable (either through inclusion of alcohol drinkers or exclusion of nondrinkers). Effect estimates were pooled using inverse variance-weighted, random-effects meta-analysis (DerSimonian and Laird method)^42^ and stratified according to whether they reported associations with prevalent or incident OAG. Odds ratios (ORs) and rate ratios (RRs) were pooled in the final meta-analysis. A method for OR to RR conversion has been proposed,^43^ but requires a baseline OAG risk, which was not available for every study, and is further complicated by the conversion of adjusted effect estimates. This method does, however, confirm that the OR is a close approximation of the RR, especially when baseline risk is <10% (the rare disease assumption) and effect estimates are small. Sensitivity analyses exploring the effect estimate derived from ORs and RRs separately were also performed.

Subgroup analyses to investigate the effects of study design (cross-sectional, case-control, cohort) and study location/population (European/North American, African/Black American, Asian) on overall effect estimates were also performed. In addition, a number of post hoc sensitivity analyses were conducted to assess the robustness of pooled estimates. These included (1) further restriction of analysis to (a) only studies with POAG as the outcome, (b) only studies with multivariable adjustment for ≥ 5 covariables; (2) only studies reporting an effect estimate as (a) an OR, (b) an RR; (3) expanding analysis to (a) all studies with a multivariable effect estimate regardless of reference exposure group, (b) all studies included in the systematic review; (4) exclusion of studies assessed as having “critical” risk of bias; and (5) analysis of effect estimates from only the highest alcohol exposure level of each included study.

Dose–response meta-analysis was not considered appropriate given the significant heterogeneity in study design and exposure definition, as well as the small number of studies reporting multiple exposure levels.

Heterogeneity of effect estimates across studies and the effect of study heterogeneity on the pooled effect estimate were assessed using the *Q* statistic and the *I*^*2*^ statistic, respectively.^44^ The *I*^*2*^ statistic was interpreted according to guidelines suggested by the Cochrane Collaboration: 0%–40% (might not be important), 30%–60% (may represent moderate heterogeneity), 50%–90% (may represent substantial heterogeneity), and 75%–100% (considerable heterogeneity).^45^ Publication bias was assessed graphically using a funnel plot and by means of the Egger^46^ and Begg^47^ tests. The trim and fill method, using the linear estimator *L*_*0*_, was used to test and adjust for funnel plot asymmetry as an additional post hoc sensitivity analysis.^48^ All analyses were conducted in Stata version 16.0 (StataCorp LLC) using the *meta* program.

The overall certainty of the evidence was assessed using the GRADE framework.^49^ Findings from the risk of bias assessment were incorporated into the GRADE assessment using the methods described by Morgan et al.^40^

## Results

### Study Identification and Selection

A total of 5201 articles were identified from the initial database search (1231 from PubMed, 2338 from Embase, 1632 from Scopus). After removal of duplicates, 3289 potentially eligible articles remained for title and abstract review. Of these, 120 articles underwent full text review, and 29 contained results pertinent to our study question. Twelve studies from duplicate study populations were excluded during the full text review process (all for incorrect exposure or outcome definitions). One further cross-sectional study^50^ was included in the IOP analysis but excluded from the OAG analysis, because a second study from the same population^29^ provided prospective data with greater adjustment for confounding variables. A further 5 articles^23^^,^^24^^,^^27^^,^^31^^,^^51^ were identified from a reference list search of all included studies and previous reviews for a total of 34 articles included in the systematic review. This included 8 studies with IOP as the outcome, 24 with OAG as the outcome, and 2 with both IOP and OAG as outcomes. Funding and conflict of interest statements for all included studies are presented in Appendix B (available at www.aaojournal.org).

**Figure 1:**
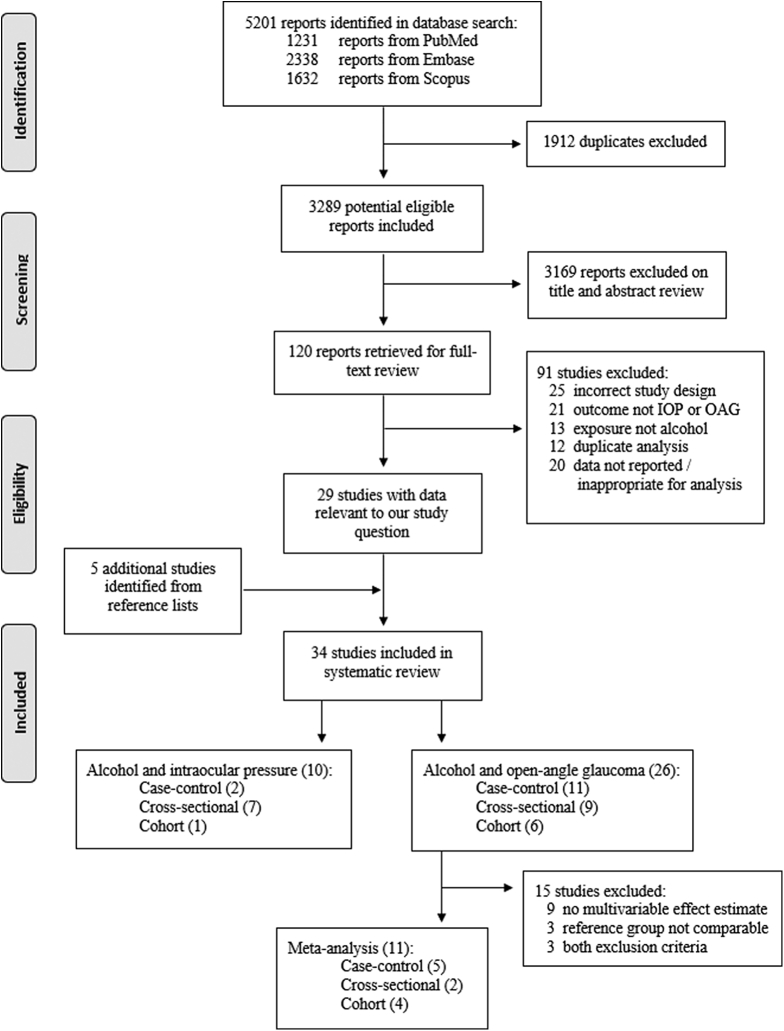
Preferred Reporting Items for Systematic Reviews and Meta-Analyses flow diagram outlining the study selection process. IOP = intraocular pressure; OAG = open-angle glaucoma.

Eleven studies reporting an association between alcohol and OAG met the criteria for meta-analysis. The full identification, screening, and selection process is detailed in Figure 1, in keeping with the Preferred Reporting Items for Systematic Reviews and Meta-Analyses guidelines.

### Characteristics and Results of Studies

#### Intraocular Pressure

The characteristics and main results of the 10 studies reporting an association between alcohol and IOP are summarized in Table 1. This included 6 studies (5 cross-sectional,^17^, ^18^, ^19^, ^20^^,^^22^ 1 prospective cohort^52^) with IOP as a continuous outcome and 4 studies (2 cross-sectional,^50^^,^^53^ 2 case-control^16^^,^^21^) with OHT as an outcome, comprising a total of 27 452 participants. Ocular hypertension was defined as IOP > 21 mmHg with no features of glaucomatous optic neuropathy by all studies using this as an outcome measure. Intraocular pressure was measured by applanation tonometry in 7 studies^16^^,^^18^^,^^19^^,^^21^^,^^22^^,^^50^^,^^52^ and noncontact tonometry in 3 studies.^17^^,^^20^^,^^53^ All studies limited their analyses to participants without glaucoma or stratified outcomes by glaucoma status. Alcohol intake was assessed through a standardized interview^16^, ^17^, ^18^, ^19^, ^20^, ^21^, ^22^^,^^50^^,^^53^ or a semiquantitative food frequency questionnaire.^52^

**Table 1:** Summary of Studies Reporting an Association between Alcohol Use and Intraocular Pressure Included in Systematic Review

Author (Year)	Location (Study)	Design	Population	Size	Outcome Measure	Result and Effect Estimate	Adjustments (Exclusions)
**Intraocular pressure**							
Lin (2005)	Taiwan∗	CS	≥65 yrs	1292	NCT	Current and former alcohol use positively associated with IOP (+0.1 mmHg).	Age, sex, SBP, DM (glaucoma)
Ramdas (2011)	Netherlands^†^	C	≥55 yrs	3939	AT	Alcohol intake (g/day) not associated with IOP in men or women for any alcohol type (beer, wine, liquor, sherry).	Age, IOP treatment (OAG)
Song (2020)	South Korea^‡^	CS	≥20 yrs	6504	AT	Alcohol use 2–3 times/wk (+0.6 mmHg) and ≥4 times/wk (+0.7 mmHg) associated with higher IOP in men without glaucoma (*P*_trend_ = 0.01). Positive association in women with glaucoma consuming ≥4 times/wk (+2.8 mmHg).	Age, sex, BMI, smoking, DM, HPT, cholesterol (ocular surgery or disease, treated glaucoma, non-OAG glaucoma, abnormal LFT)
Weih (2001)	Australia^§^	CS	≥40 yrs	4576	AT	Previous, but not current, use of alcohol negatively associated with IOP (−<0.1 mmHg) in participants without glaucoma.	Rural residence, iris color, vitamin E intake, SE (treated glaucoma)
Wu (1997)	West Indies^ǁ^	CS	40–84 yrs	3752	AT	Use of alcohol in the past year positively associated with IOP (+0.1 mmHg).	Age, sex, complexion, BMI, SBP, DM, smoking, PR, family history, ocular surgery or infection, examination season (glaucoma)
Yoshida (2003)	Japan	CS	29–79 yrs	569	NCT	Never or seldom alcohol use (-1.4 mmHg) and use several times per month (-0.8 mmHg) associated with lower IOP compared with daily use (*P*_trend_ < 0.001) in men but not women.	BMI, SBP, smoking, exercise, coffee (HPT, OHT, glaucoma)
**Ocular hypertension**							
Doshi (2008)	USA^¶^	CS	≥40 yrs	5843	AT	Alcohol use: categorical (ex-/partial, current/heavy), g/wk (<40, 40–104, ≥105), type (wine, beer, liquor) not associated with OHT.	Age, Native American ancestry, employment status (glaucoma)
Lee (2019)	South Korea	CS	Males, <65 yrs, BMI ≥25	479	NCT	Any alcohol use not associated with OHT in participants with and without alcohol-induced flushing reaction (see “Discussion”). Evidence of effect mediation by total weekly alcohol intake.	Age, BMI, SBP, smoking, DM, cholesterol, CVD, thyroid function, ocular surgery (glaucoma)
Leske (1996)	USA^#^	CC	≥40 yrs	298	AT	Ever use of alcohol associated with OHT, OR 2.32 (95% CI, 1.15–4.69).	Age, sex, family history, HPT, smoking (glaucoma)
Seddon (1983)	USA	CC	Adults, age range not defined	200	AT	No liquor intake (compared with daily intake) associated with OHT, OR 3.8 (95% CI, 1.4–10.4) with stronger association noted in men (OR 9.2). No association with other alcohol types.	Age, sex, family history, myopia, income, BP, stress, ocular surgery (glaucoma)

∗Shihpai Eye Study, ^†^Rotterdam Study, ^‡^Korea National Health and Nutrition Examination Survey, ^§^Melbourne Visual Impairment Project, ^ǁ^Barbados Eye Study, ^¶^Los Angeles Latino Eye Study, ^#^Long Island Glaucoma Case-Control Study Group.

AT = applanation tonometry; BMI = body mass index; BP = blood pressure; C = cohort; CC = case-control; CI = confidence interval; CS = cross-sectional; CVD = cardiovascular disease; DM = diabetes mellitus; HPT = hypertension; IOP = intraocular pressure; LFT = liver function test; OAG = open-angle glaucoma; OHT = ocular hypertension; NCT = noncontact tonometry; OR = odds ratio; PR = pulse rate; SBP = systolic blood pressure; SE = spherical equivalent.

Alcohol use was positively associated with IOP in 2 studies,^17^^,^^19^ although the absolute difference between drinkers and nondrinkers (0.1 mmHg in both studies) was small. A further 2 studies found positive linear associations between alcohol intake and IOP in men, but not women, without glaucoma (IOP difference of 0.7–1.4 mmHg between highest intake group and no intake group).^18^^,^^20^ In one of these studies, consumption of alcohol > 4 times/week in women with glaucoma was associated with higher IOP (+2.8 mmHg) compared with nondrinkers, but with no evidence of linear trend.^18^ Alcohol intake was not associated with IOP in 1 study^52^ and negatively associated (IOP difference <0.1 mmHg) in previous, but not current, drinkers in another.^22^

Alcohol use was associated with OHT in 1 included study,^16^ with no association reported in a further 2 studies.^50^^,^^53^ A protective association with the use of liquor (but not other alcohol types) was found in the final study exploring this association.^21^

Within each outcome subgroup (IOP and OHT), further heterogeneity in exposure definition (including both continuous and categorical alcohol intake measures, as well as stratifications by sex, glaucoma status, alcohol type, and flushing reaction) resulted in a limited number of studies with sufficiently similar results to allow for meaningful meta-analysis of the association between alcohol use and IOP.

#### Open-Angle Glaucoma

Twenty-six studies reported an association between alcohol use and OAG. The full case ascertainment criteria for these studies are presented in Table S2 (available at www.aaojournal.org). Of these, 15 studies (comprising 41 123 participants) were excluded from meta-analysis due to lack of a multivariable effect estimate (n = 9), a reference exposure group that was not comparable (n = 3), or both (n = 3). The characteristics and main results of these excluded studies are presented in Table S3 (available at www.aaojournal.org). In summary, of the excluded studies, 1 case-control study found a harmful association between alcohol and OAG,^54^ 11 studies (7 cross-sectional,^51^^,^^55^, ^56^, ^57^, ^58^, ^59^, ^60^ 2 case-control,^61^^,^^62^ 2 prospective cohort^52^^,^^63^) found no association, and 2 case-control studies found protective associations.^64^^,^^65^ A final case-control study reported a protective association in participants of African descent but a harmful association in participants of European descent.^66^

The characteristics of the 11 studies (2 cross-sectional,^23^^,^^24^ 5 case-control,^16^^,^^25^, ^26^, ^27^, ^28^ 4 cohort^29^, ^30^, ^31^^,^^67^), comprising 173 058 participants, included in the meta-analysis of alcohol use and OAG are presented in Table 4. Seven reported associations with prevalent OAG,^16^^,^^23^, ^24^, ^25^, ^26^, ^27^, ^28^ and 4 reported associations with incident OAG.^29^, ^30^, ^31^^,^^67^ Primary open-angle glaucoma was the outcome variable in 7 of the studies.^24^, ^25^, ^26^^,^^28^^,^^30^^,^^31^^,^^67^ The main results and effect estimates of these studies are presented in Table 5. Five studies reported multiple alcohol exposure levels, and a single pooled effect estimate across all levels was calculated for use in meta-analysis.^26^^,^^28^, ^29^, ^30^^,^^67^ Overall, 10 studies reported no association between any alcohol use and OAG,^16^^,^^23^, ^24^, ^25^, ^26^, ^27^, ^28^, ^29^, ^30^, ^31^ with only one large cohort study of Black women reporting a harmful association.^67^ Although there was a suggestion of a dose–response effect in those studies reporting ordinal alcohol exposure levels,^26^^,^^28^^,^^30^^,^^67^ no study-specific test for trend reached statistical significance. Only 3 of these studies reported comparable, quantifiable alcohol exposure levels,^28^^,^^30^^,^^67^ and further heterogeneity in study design (1 cross-sectional, 2 longitudinal) precluded meaningful dose–response meta-analysis. There was also no evidence of an association by alcohol type^30^ or OAG phenotype (normal-tension or high-tension)^24^^,^^26^ in the included studies.

**Table 4:** Characteristics of Studies Included in the Meta-analysis of the Association between Alcohol Use and Open-Angle Glaucoma

Author (Year)	Location (Study)	Design	Population	Size (Cases)	Exposure Measure	Outcome	Adjustment (Covariates or Matched Variables)
**Prevalent OAG**							
Bikbov (2020)	Russia (Russian Ural Eye and Medical Study)	CS	≥40 yrs	5545 (177)	IAQ	OAG	Age
Bonomi (2000)	Italy (Egna-Neumarkt Study)	CS	≥40 yrs	4147 (60)	IAQ	POAG	Sex
Charliat (1994)	Netherlands	CC	≥40 yrs	350 (175)	SAQ	POAG	Age, sex, type of health care
Chiam (2018)	Singapore (Singapore Chinese Eye Study)	CC	≥40 yrs	3499 (2788)	IAQ	POAG	Age, sex, IHD, stroke, HPT, hyperlipidemia, DM, migraine, smoking, family history, myopia, IOP, CCT
Leske (1996)	USA (Long Island Glaucoma Case-Control Study Group)	CC	≥40 yrs	312 (190)	IAQ	OAG	Age, sex, family history, HPT, smoking
Leske (2001)	West Indies (Barbados Family Study of Open-Angle Glaucoma)	CC	≥25 yrs	286 (219)	IAQ	OAG	Age, sex, sibling relation
Renard (2013)	France (Photograf Study)	CC	≥40 yrs	678 (339)	IAQ	POAG	Age, sex, duration of disease
**Incident OAG**							
Jiang (2012)	USA (Los Angeles Latino Eye Study)	C	≥40 yrs	3772 (87)	IAQ	OAG	Age, IOP, AL, lack of vision insurance, WHR, CCT, smoking, SBP, OPP, DM, cataract surgery, family history
Kang (2007)	USA (Nurses Health Study & Health Professionals Follow-Up Study)	C	≥40 yrs	120379 (856)	SQFFQ	POAG	Age, family history, Black heritage, HPT, DM, BMI, smoking, physical activity, caffeine, caloric intake
Pan (2017)	China (Yunnan Minority Eye Study)	C	≥50 yrs	1520 (19)	IAQ	POAG	Age, sex, IOP, CCT, AL, myopia, BMI, education, HPT, DM, smoking
Wise (2011)	USA (Black Women’s Health Study)	C	Female, 21–69 yrs	32570 (366)	SAQ	POAG	Age, questionnaire cycle, education, smoking, HPT, physical activity, energy intake, BMI

AL = axial length; BMI = body mass index; C = cohort; CC = case-control; CS = cross-sectional; CCT = central corneal thickness; DM = diabetes mellitus; HPT = hypertension; IAQ = interviewer-administered questionnaire; IHD = ischemic heart disease; IOP = intraocular pressure; OAG = open-angle glaucoma; OPP = ocular perfusion pressure; POAG = primary open-angle glaucoma; SAQ = self-administered questionnaire; SBP = systolic blood pressure; SQFFQ = semi-quantitative food frequency questionnaire; WHR = waist:hip ratio.

**Table 5:** Results and Effect Estimates of Studies Included in the Meta-analysis of the Association between Alcohol Use and Open-Angle Glaucoma

Author (Year)	Reference Group	Exposure Level/s	Effect Estimate (95% CI)	Pooled Effect Estimate (95% CI)	Additional Results
**Prevalent OAG**					
Bikbov (2020)	No consumption	Any consumption	OR 1.81 (0.99–3.31)	N/A	
Bonomi (2000)	No consumption	Any consumption	OR 1.40 (0.80–2.20)	N/A	No association when stratified by HTG (>21 mmHg) or NTG (≤21 mmHg).
Charliat (1994)	No consumption	Any consumption	OR 1.00 (0.57–1.73)	N/A	
Chiam (2018)	No consumption	<2 days/wk ≥2 days/wk	OR 1.08 (0.51–2.32) OR 1.27 (0.53–3.03)	OR 1.16 (0.65–2.05)	No association when stratified by HTG or NTG. No association with alcohol type in univariable analyses.
Leske (1996)	No consumption	Any consumption	OR 1.22 (0.66–2.24)	N/A	No association when OAG cases compared with OHT controls.
Leske (2001)	No consumption	Any consumption	OR 0.80 (0.34–1.88)	N/A	
Renard (2013)	0 drinks/day	0–1 drinks/day 1–2 drinks/day 2–3 drinks/day >3 drinks/day	OR 0.85 (0.51–1.42) OR 0.75 (0.42–1.34) OR 1.35 (0.66–2.74) OR 0.81 (0.29–2.31)	OR 1.14 (0.93–1.40)	*P*_trend_ > 0.10. No association with binge drinking (≥5 drinks/occasion).
**Incident OAG**					
Jiang (2012)	No consumption	Previous consumption Current consumption	OR 1.59 (0.95–2.64) OR 0.76 (0.28–2.06)	OR 1.36 (0.87–2.15)	
Kang (2007)	0 g/day	1–9 g/day 10–19 g/day 20–29 g/day ≥30 g/day	RR 0.99 (0.83–1.19) RR 0.96 (0.76–1.22) RR 0.95 (0.68–1.33) RR 0.71 (0.49–1.04)	RR 0.94 (0.83–1.07)	*P*_trend_ = 0.09. No association with alcohol type.
Pan (2017)	No consumption	Any consumption	OR 2.40 (0.80–7.50)	N/A	
Wise (2011)	0 drinks/wk	1–6 drinks/wk ≥7 drinks/wk	RR 1.28 (1.01–1.62) RR 1.60 (1.06–2.43)	RR 1.35 (1.10–1.66)	*P*_trend_ = 0.17. Stronger associations noted in women <50 yrs. Harmful association in current (RR, 1.35, 95% CI, 1.05–1.73) but not former drinkers. No association with total years of alcohol drinking.

CI = confidence interval; HTG = high-tension glaucoma; OAG = open-angle glaucoma; OHT = ocular hypertension; NTG = normal-tension glaucoma; N/A = not available; OR = odds ratio; RR = rate ratio.

#### Meta-analysis

**Figure 2:**
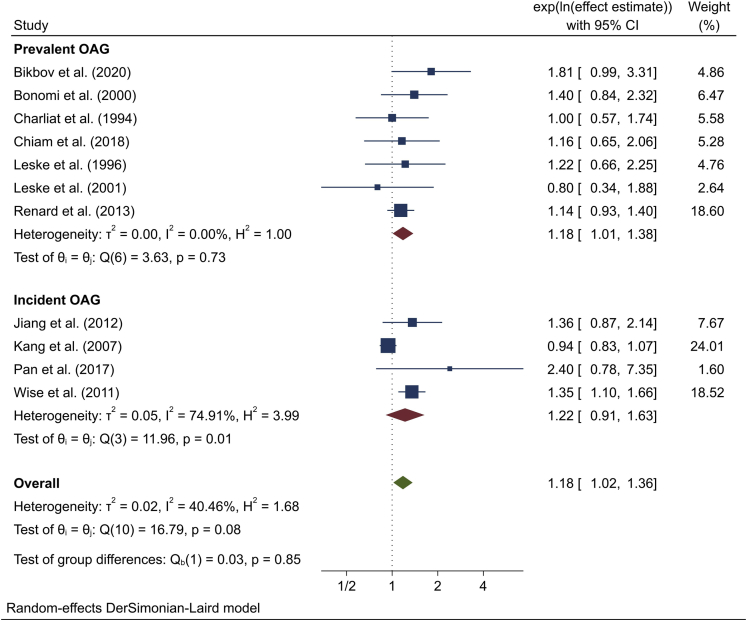
Meta-analysis of the association between alcohol use and open-angle glaucoma (OAG). The confidence intervals (CIs) in this figure may not be equivalent to those presented in Table 5 due to rounding differences in meta-analysis software.

Meta-analysis of effect estimates from the 11 included studies showed that any consumption of alcohol was significantly associated with OAG (overall effect estimate 1.18; 95% CI, 1.02–1.36; *P* = 0.03; *I*^*2*^ = 40.5%) when compared with no consumption (Fig 2). Similar effect sizes were obtained for both prevalent (1.18; 95% CI, 1.01–1.38; *I*^*2*^ = 0.0%) and incident (1.22; 95% CI, 0.91–1.63; *I*^*2*^ = 74.9%) OAG, with no evidence of heterogeneity between groups (*P* = 0.85).

The strongest effect estimates were obtained for cross-sectional studies (1.56; 95% CI, 1.06–2.29; n = 2) and studies from Asia (1.53; 95% CI, 1.03–2.25; n = 3), although there was no evidence of heterogeneity by study design (*P* = 0.30) or study location/population (*P* = 0.20). Effect estimates derived from various sensitivity analyses did not differ substantially from the main result (range, 1.15–1.21), although loss of participant or study numbers often resulted in wider confidence intervals and loss of statistical significance. A slightly stronger effect was obtained from meta-analysis of only those studies reporting results as an OR (effect estimate 1.21; 95% CI, 1.05–1.40). There was significant heterogeneity (*P* < 0.01) between studies reporting a univariable effect estimate (0.86; 95% CI, 0.78–0.95), which suggest a protective effect, and those with a multivariable effect estimate (1.18; 95% CI, 1.04–1.34), which instead point to a harmful effect, included in this systematic review. Full details of subgroup and sensitivity analyses are reported in Table 6.

**Table 6:** Meta-analysis of the Association between Alcohol Use and Open-Angle Glaucoma: Subgroup and Sensitivity Analyses

Description (Number of Studies in Meta-analysis)	Effect Estimate (95% CI)		*P*_heterogeneity_
**Subgroup analyses**			
Study design			0.30
Case-control (5)	1.12	(0.94–1.33)	
Cross-sectional (2)	1.56	(1.06–2.29)	
Cohort (4)	1.22	(0.91–1.63)	
Study location/population			0.20
European/North American (6)	1.06	(0.93–1.21)	
African/Black American (2)	1.23	(0.84–1.82)	
Asian (3)	1.53	(1.03–2.25)	
**Sensitivity analyses**			
(1a) Include only studies with POAG as outcome (7)	1.15	(0.97–1.36)	
(1b) Include only studies with adjustment for ≥5 covariables (6)	1.19	(0.95–1.50)	
(2a) Include only studies with odds ratio as effect estimate (9)	1.21	(1.05–1.40)	
(2b) Include only studies with rate ratio as effect estimate (2)	1.12	(0.78–1.59)	
(3a) Include studies with different baseline reference category (14)	1.18	(1.04–1.34)	
(3b) Include all studies from systematic review			<0.01
Univariable effect estimate (12)	0.86	(0.78–0.95)	
Multivariable effect estimate (14)	1.18	(1.04–1.34)	
(4) Exclude studies with “critical” risk of bias (9)	1.18	(1.01–1.39)	
(5) Include only effect estimates from highest exposure level (11)	1.20	(0.97–1.50)	

CI = confidence interval; POAG = primary open-angle glaucoma.

Although neither the Begg (*P* = 0.38) nor Egger (*P* = 0.51) tests suggested publication bias, there was an indication of funnel plot asymmetry with more studies appearing to the right of the pooled estimate. Stratified funnel plots showed symmetry of studies reporting associations with prevalent OAG, with the observed asymmetry arising from studies of incident OAG (Fig S3, available at www.aaojournal.org). Trim and fill analysis resulted in the imputation of 2 hypothetical studies both situated to the left of the pooled estimate (Appendix C, available at www.aaojournal.org). The updated effect estimate (based on 11 observed and 2 imputed studies) was slightly attenuated (1.14; 95% CI, 0.99–1.32).

#### Risk of Bias and GRADE Assessment

Assessment of study quality revealed residual confounding, exposure classification, and departures from exposure to be the greatest risks of bias across all included studies (Fig S4, available at www.aaojournal.org). Residual confounding was identified as a domain of particular concern, with most studies at “serious” or “critical” risk of bias. Overall, 2 studies were deemed to be at “critical” risk,^24^^,^^27^ with only one study achieving a “moderate” risk of bias.^30^

Although these risks varied between the included studies, assessment of study quality was not used as a weighting tool or exclusion criterion for the final meta-analysis. A post hoc sensitivity analysis excluding studies with “critical” risk of bias, however, did not materially change the overall effect estimate.

The overall GRADE certainty of evidence assessment was “very low.” Observational studies are assigned an initial “low” level of evidence, and this was further downgraded for study limitations (risk of bias) and inconsistency (heterogeneity) in the evidence base. The assessment was upgraded 1 level because sensitivity analysis suggested that the plausible effect of residual confounding would be to strengthen the overall effect. Full details of the GRADE assessment are shown in Table 7.

**Table 7:** GRADE Assessment of Studies Included in Meta-analysis of Alcohol Use and Open-Angle Glaucoma

		Factors That Can Reduce the Quality of the Evidence					Factors That Can Increase the Quality of the Evidence			
Number of Studies	Design∗	*Study Limitations*†	*Inconsistency*‡	*Indirectness*§	*Imprecision*‖	*Publication Bias*¶	*Large Magnitude of Effect*#	*Dose*–*response Effect*	*Plausible Effect of Residual Confounding*∗∗	Overall Quality of Evidence
11 (173 058 participants)	Observational	High	Present	None	None	None	None	None	Present	⊕○○○
*Evidence*	*Low*	*−1*	*−1*	*0*	*0*	*0*	*0*	*0*	*+1*	Very low

∗ Observational studies are assigned a default “low” level of evidence, which can then be downgraded or upgraded further according to various factors.

† Assessed using a Risk of Bias tool designed for nonrandomized studies of exposures (Fig S4, available at www.aaojournal.org). Downgraded 1 level due to “critical” limitation in 1 domain.

‡ Criteria for significant inconsistency of results were *I*^*2*^ > 50% or *P* < 0.10 for the chi-square test of heterogeneity.

§ All studies assessed the association between self-reported alcohol consumption and a diagnosis of open-angle glaucoma.

‖ Not downgraded due to large sample size and 95% confidence intervals excluding no effect.

¶ The possibility of publication bias is not excluded but it was not considered sufficient to downgrade the quality of evidence.

# Defined as effect estimate >2.0 or <0.50, based on direct evidence with no plausible confounders.

∗∗ Sensitivity analysis revealed significant heterogeneity between studies reporting unadjusted and adjusted effect estimates, with the suggestion that further adjustment would result in a stronger effect.

## Discussion

This study provides a systematic review of the current evidence for the association of habitual alcohol consumption with IOP and OAG. Although numerous identified studies provided quantitative estimates for these associations, few were designed specifically to investigate these relationships. Consequently, there is considerable heterogeneity in the current evidence base, and most results are limited to a simple binary comparison (drinkers vs. nondrinkers), without further interrogation or sensitivity analyses. This has important implications for direct comparability and meta-analytical approaches, and although we attempted to account for these limitations in our analyses as far as possible, any pooled quantitative estimates should be viewed in the context of the largely questionable data strength of the underlying studies. Furthermore, the pooled effect estimate for the association with OAG was small and of borderline statistical significance. Although estimates were largely consistent across sensitivity analyses, the statistical evidence for these results was generally weaker, and it is conceivable that further adjustment for residual confounding factors would render our main finding nonsignificant. Therefore, this meta-analysis should not in itself be considered strong evidence for a harmful association, but rather as an analytical approach to the synthesis of a widely heterogeneous evidence base that is best considered alongside the qualitative appraisal of the evidence that follows.

### Physiology

The acute ocular hypotensive effects of alcohol have been known for at least 50 years,^12^ although the precise physiologic mechanism for the IOP reduction remains unclear. Hypotheses include a transient osmotic effect after alcohol consumption, suppression of antidiuretic hormone with a reduction in net ocular water movement, and a direct inhibitory effect on the secretory cells of the ciliary epithelium.^9^^,^^21^^,^^30^ This effect appears to be dose-dependent; a nonsignificant IOP reduction was noted after ingestion of < 10 g alcohol,^68^ with absolute reductions of 1 to 4 mmHg after 10 to 30 g,^7^^,^^8^^,^^11^^,^^13^ and up to 6 mmHg with doses approaching 40 g,^12^ but is seemingly independent of alcohol concentration or total fluid volume. Equal quantities of alcohol administered in different concentrations (as beer or whiskey) produced similar IOP-lowering effects,^12^ whereas administration of equal volumes of beer and water produced opposite effects.^14^ Little to no effect on IOP was noted when alcohol was administered together with antidiuretic hormone or to individuals with abnormal posterior pituitary gland function.^9^ The peak ocular hypotensive effect is usually noted at 1 to 3 hours after ingestion,^8^, ^9^, ^10^, ^11^, ^12^, ^13^^,^^68^ depending on the dose and may last up to 5 hours.^12^ Ocular hypotension can be maintained through repeated oral or intravenous alcohol doses,^9^ and a more pronounced effect is noted in eyes with a higher baseline IOP. Absolute reductions of 12 to 30 mmHg have been reported in glaucomatous eyes.^9^^,^^12^ In addition to lowering IOP, alcohol also results in a significant increase in retrobulbar and optic nerve head blood flow^13^^,^^15^ and retinal artery diameter^11^ but does not appear to have an effect on ocular perfusion pressure.^11^^,^^15^

### Intraocular Pressure

Although the short-term physiologic effects of alcohol have been well established in experimental studies, this relationship does not translate to population-based studies. Observational studies included in this systematic review generally show either a small positive association or no association between alcohol use and IOP^17^, ^18^, ^19^, ^20^^,^^52^ or OHT,^16^^,^^50^^,^^53^ but this in itself is not a consistent result.^21^^,^^22^ One further study excluded from this review also reported no association between alcohol use and IOP but did not present specific data for this finding.^55^ In addition, absolute IOP differences between drinkers and nondrinkers are often small (maximum difference in participants without glaucoma +1.4 mmHg), although most studies excluded participants with glaucoma from analysis. Given the strong association between IOP and glaucoma, exclusion of these individuals may have altered the IOP distribution in the remaining participants, potentially attenuating any observed IOP difference. Women with untreated OAG consuming alcohol ≥ 4 times/week were found to have a higher IOP (+2.8 mmHg) than nondrinkers in a South Korean study,^18^ but this relationship was not apparent in men nor was it demonstrated in an Australian study that also included participants with glaucoma in analysis.^22^ Evidence of stronger effects and linear trend between alcohol intake and IOP also appear to be restricted to men, but this finding may be explained by a smaller number of female drinkers in these studies.^18^^,^^20^

There are numerous considerations when interpreting the available evidence for the association between alcohol use and IOP. If alcohol is not consumed at a frequency regular enough to result in sustained ocular hypotension or in the hours preceding IOP measurement, this physiologic effect may not be apparent. In addition, the direct short-term effects of alcohol may be outweighed by potential indirect or long-term IOP-raising effects. For example, both systolic and diastolic blood pressure are positively associated with alcohol consumption and IOP.^4^^,^^69^^,^^70^ Although most studies adjusted for blood pressure or hypertension in their analyses,^16^, ^17^, ^18^, ^19^, ^20^, ^21^^,^^53^ it is possible that any observed association may be due to residual confounding by various vascular (or other) risk factors. Alternatively, alcohol may have a true direct effect on IOP, although small and mediated via uncertain pathophysiologic mechanisms.

### Open-Angle Glaucoma

The earliest report of a harmful association between alcohol and OAG arose from the Framingham Eye Study in 1980 when formal diagnostic criteria for glaucoma were not yet established.^71^ It was found that alcohol intake was associated with various definitions of OAG, largely based on visual field defects, but also with definitions encompassing IOP and cup-disc ratios. Subsequently, numerous observational studies conducted during the 1980s and 1990s reported no association between alcohol use and OAG.^55^^,^^62^^,^^72^, ^73^, ^74^ A number of these earlier studies,^72^, ^73^, ^74^ as well as more recent studies,^75^, ^76^, ^77^ however, did not report specific data or effect estimates for this association and were therefore excluded from this systematic review. Indeed, the majority of studies (10/11) included in the final meta-analysis reported no association between alcohol intake and prevalent or incident OAG.^16^^,^^23^, ^24^, ^25^, ^26^, ^27^, ^28^, ^29^, ^30^, ^31^ Only when these results are meta-analyzed does a significant harmful association become apparent.

Prospective evidence from the 2 largest studies exploring the association between alcohol intake and OAG report seemingly contradictory findings. Wise et al^67^ found a harmful association in a large cohort study of Black women (Black Women’s Health Study [BWHS]), especially in those consuming ≥ 7 drinks/week (RR, 1.60; 95% CI, 1.06–2.43). In contrast, Kang et al^30^ found that consumption of >30 grams of alcohol per day appeared to be protective for incident POAG (OR, 0.71; 95% CI, 0.49–1.04) in the Nurses’ Health Study and Health Professionals Follow-Up Study (NHS/HPFS), although this result did not reach statistical significance. Various important differences between these 2 study populations need to be considered when interpreting this result. First, participants in the NHS/HPFS were approximately 20 years older than those in the BWHS. Given the significant association between alcohol intake and all-cause mortality,^6^^,^^78^ competing events in the NHS/HPFS may have contributed to an underestimation of POAG risk, especially in older participants with the highest alcohol intake. However, because participants tended to be middle-aged (∼60 years) and moderate drinkers, a group not at increased risk for all-cause mortality,^78^ this is unlikely to be a major contributory factor. Second, the NHS/HPFS consisted entirely of health professionals, a group that is likely to differ substantially from the general population in various ways, including in factors related to alcohol-intake behaviors, reporting of alcohol consumption and general health status. Finally, the BWHS consisted entirely of Black participants, but this group made up only 1% of participants in the NHS/HPFS. Likewise, women represented all participants in the BWHS but 65% of those in the NHS/HPFS. It is possible that any risk may be mediated by both race and sex, but there is currently no evidence to support this explanation. Only one small case-control study reported effect estimates stratified by race,^62^ and there was no suggestion of heterogeneity by study population/location in this meta-analysis. Likewise, findings from the NHS/HPFS were consistent across sexes, and sex was not found to be a significant factor in the only study reporting stratified results included in this systematic review.^55^

The overall effect estimate was robust across all sensitivity analyses with the exception of studies reporting an univariable effect estimate, in which a significant protective association was observed. We hypothesize that this may be due to the confounding effect of variables such as age and socioeconomic status, which have associations with both alcohol intake and the occurrence or diagnosis of glaucoma.^79^^,^^80^

There are a number of possible explanations for the observed association between alcohol use and OAG in this meta-analysis, and these should be considered within the context of the weakness and heterogeneity of the supporting evidence. Alcohol may be directly implicated in OAG risk, although the exact pathophysiologic mechanisms are not clear. Chronic alcohol use can lead to significant peripheral neuropathy, and the proposed underlying mechanisms may play a similar role in glaucomatous optic neuropathy.^81^ These include oxidative stress leading to free radical damage to nerves, activation of the sympathoadrenal and hypothalamo–pituitary–adrenal axes, nutritional deficiencies (especially thiamine), and direct toxic and proinflammatory effects. Alternatively, alcohol may indirectly influence OAG risk through its association with a number of neurodegenerative and cardiovascular diseases, and it is possible that residual confounding effects may be responsible for the observed association. This systematic review also suggests a positive association between alcohol use and IOP, which may further contribute to OAG risk.

### Dose–Response Effects

An important consideration in the interpretation of observational studies of environmental or lifestyle exposures is evidence of a dose–response effect that, if present, supports the hypothesis of a causal relationship between associated variables. Alcohol intake has a linear, logarithmic, or J-shaped association with a multitude of disease outcomes.^4^^,^^6^ Dose-dependent associations between alcohol and IOP were demonstrated in men without glaucoma in 2 studies,^18^^,^^20^ but this was not a consistent finding. Although there was a suggestion of both harmful^26^^,^^67^ and protective^30^ dose-dependent linear relationships between alcohol intake and OAG, statistical significance was not demonstrated in any study included in this systematic review,^28^^,^^30^^,^^67^ and formal dose-dependent meta-analysis was not performed. Furthermore, there was no consistent finding regarding the association in current and previous alcohol drinkers.^29^^,^^67^ Future research should aim to better define the dose–response relationship between alcohol and various glaucoma-related outcomes and traits, including the possibility of a nonlinear relationship.

### Alcohol Type

Aside from their ethyl alcohol content, there are considerable differences in the constituents and global consumption patterns of the wide variety of alcoholic beverages available.^6^^,^^82^ Therefore, it is important to consider the possible confounding role that these factors may play when exploring any associations with alcohol consumption. Of particular interest are the polyphenols, a group of compounds with anti-inflammatory and antioxidant properties, which are found in high levels in red wine and may play a promising role in improving visual function and slowing visual field loss in patients with OHT and glaucoma.^83^ However, alcohol type,^26^^,^^30^^,^^52^ and specifically red wine,^30^ was not found to be associated with OAG in any study included in this systematic review. One case-control study reported a protective association between daily liquor intake (but not intake of any other alcohol type) and OHT,^21^ but this finding has not been reproduced in other studies.

### Glaucoma and Related Outcomes

OCT measurement of the peripapillary and macular retinal nerve fiber layer (RNFL) plays an important role in the diagnosis and management of glaucoma. Although alcohol intake was found not to be associated with peripapillary RNFL thickness in the EPIC-Norfolk Eye Study,^84^ higher levels of alcohol consumption (women: > 10 g/day; men: > 20 g/day) were found to be associated with peripapillary RNFL thinning in the Gutenberg Health Study.^85^ In addition, high levels of alcohol consumption have been found to be associated with thinning of various macular inner retinal parameters, particularly the ganglion cell-inner plexiform layer (GC-IPL), in both the UK Biobank^86^ and Beaver Dam Offspring^87^ studies. This association is not only limited to population-based studies; alcohol intake was associated with GC-IPL thinning in patients with known POAG in a South Korean study.^88^

Although these findings suggest that alcohol may play a role in glaucoma severity and progression, there is limited other evidence in this regard. Alcohol use has not been associated with visual field defect deterioration in known glaucoma patients,^89^ progression from POAG suspect to definite POAG,^90^ or progression to blindness in high-tension POAG.^91^ Alcohol consumption was also not found to be associated with incident self-reported glaucoma in the SUN cohort^92^ or with prevalent glaucoma in a German case-control study.^93^

### Genetic Considerations

A number of studies have explored the potential role and associations of gene–alcohol interactions with IOP and glaucoma. A particular focus has been the aldehyde dehydrogenase 2 (ALDH2) gene, which plays a central role in alcohol metabolism.^94^ The ALDH2 enzyme converts acetaldehyde, a toxic by-product of alcohol metabolism, to nontoxic acetic acid. Polymorphisms in the ALDH2 gene, which are particularly common in East Asian populations, may result in an inactive form of the ALDH2 enzyme and lead to a systemic accumulation of acetaldehyde when alcohol is consumed. Characteristic effects of ALDH2 enzyme deficiency include reduced alcohol tolerance, as well as alcohol-induced facial flushing, tachycardia, and palpitations. A South Korean study found that drinking-related facial flushing in overweight men was associated with OHT at lower levels of alcohol consumption than in nonflushers.^53^ However, ALDH2 (rs671) polymorphism was found not to be associated with peripapillary RNFL or GC-IPL thickness in patients with known POAG in another South Korean study, although gene–alcohol interactions were not analyzed.^88^ The alcohol-induced increase in retrobulbar blood flow has been shown to be more pronounced in ALDH2-deficient individuals.^15^

Nitric oxide synthase 3, an enzyme that mediates luminal smooth muscle tone and found in both trabecular meshwork and ocular vascular endothelial cells, has previously been implicated as a potential factor in the pathogenesis of OAG.^95^ However, the association between nitric oxide synthase 3 genetic variants and POAG was found not to be modified by alcohol consumption in a subsequent nested case-control study.^96^

Genetic variants of toll-like receptor 4, a transmembrane pathogen recognition receptor able to mediate the release of inflammatory cytokines, have been associated with POAG and normal-tension glaucoma in the Japanese population. Significant gene–alcohol interaction has been reported in a Chinese study, with the highest POAG risk observed in alcohol drinkers carrying a toll-like receptor 4 (rs2149356) polymorphism.^54^

The longevity-associated mitochondrial DNA 5178C polymorphism has a reported interaction with alcohol. Daily consumption in Japanese men with a mt5178C polymorphism was found to be significantly associated with higher IOP.^97^

### Study Strengths and Limitations

Based on the results of our literature search, this study represents the only systematic review and meta-analysis of the associations of alcohol consumption with IOP and OAG to date. There are a number of important factors to consider when interpreting the study results, in addition to the limitations already discussed.

As is the case with the study of most environmental exposures, evidence is limited to observational studies that have inherent weaknesses and risks of bias. Alcohol studies, in particular, are subject to further specific risks and methodological pitfalls.^98^ Although well-conducted observational studies can minimize the potential biases introduced by factors such as participant selection, residual confounding, and reverse causality, it is possible that the findings of this systematic review and meta-analysis are influenced by study-specific and systematic biases. This was apparent in the findings of the risk of bias assessment, with domains relating to residual confounding and exposure ascertainment identified as particular areas of concern. In addition to heterogeneity, this risk of bias was deemed sufficient to further downgrade the overall GRADE certainty of evidence to “very low.”

There is currently no universally accepted standard or consensus for assessing risk of bias in observational studies, and various concerns with early versions of the ROBINS-E tool have been raised.^99^ Specific criticisms include rating observational studies in comparison with an “ideal” randomized controlled trial when this is often not practically possible; failure to discriminate between studies with single or multiple risks of bias; equal weighting of all risk of bias domains; and serious limitations in determining whether confounders will bias study outcomes. Therefore, although an important consideration in any systematic review and meta-analysis, given the current limitations, as well as the subjective nature of such an assessment, risk of bias was not used as a weighting tool or exclusion criterion for the final meta-analysis. Furthermore, the presence of other limitations in the current evidence base make it unlikely that this would significantly alter the overall GRADE certainty of evidence.

Results did prove to be robust across the various sensitivity analyses, however, with the greatest risk of bias identified arising from univariable effect estimates. There was also no statistical evidence of publication bias despite a suggestion of funnel plot asymmetry. Trim and fill analysis, which detects and attempts to correct funnel plot asymmetry, resulted in slight attenuation of the overall effect estimate. It is important to note that this method is agnostic as to the reasons behind the funnel plot asymmetry and may underestimate a true positive effect if no publication bias is present.^100^ Other possible explanations for the observed asymmetry include effect size heterogeneity across studies, especially considering the difference between estimates for prevalent (*I*^*2*^ = 0.0%) and incident (*I*^*2*^ = 74.9%) OAG, and chance.

Few studies included in this systematic review were conducted specifically to explore the association between alcohol and IOP or OAG. Instead, most effect estimates are derived from studies that examined different or multiple exposures. Subsequently, our search strategy may have failed to detect similar relevant studies, especially if alcohol was not mentioned specifically in the article title, abstract, or keywords. This was the case for the 5 additional studies identified during the manual search of the reference lists of included studies and previous reviews. All studies identified in this manner were epidemiological eye studies that collected alcohol intake data in addition to numerous other baseline characteristics. Although all studies reported associations with alcohol intake, this was not the primary study focus, and all were indexed without specific reference to alcohol or related terms.

Although case ascertainment criteria for OAG were generally appropriately stringent, objective, and comparable across studies (most requiring a combination of direct visual field, optic nerve head, and angle assessment), measurement of alcohol exposure was far more variable and may have led to significant misclassification bias. Most studies based their exposure assessment on self-reported alcohol consumption from a single questionnaire that, although practical, is subject to both recall and social desirability bias. This was further complicated by variable definitions of “regular” alcohol intake as well as time periods under consideration. Even semiquantitative food frequency questionnaires, which are generally based on current or recent drinking behaviors, may not accurately reflect alcohol consumption over the life-course or drinking patterns such as binge drinking. Significant heterogeneity in categories or levels of alcohol exposure also precluded meaningful dose–response meta-analysis. This limitation in the evidence makes it difficult for health professionals to recommend a “safe dose” of alcohol consumption with regard to glaucoma risk.

## Conclusions

Findings from this study suggest that alcohol consumption is positively associated with IOP, although the absolute effect size appears small. In addition, a possible association between alcohol consumption and OAG was demonstrated. This finding should be interpreted with caution, however, given the significant methodological heterogeneity and risk of bias present in the underlying evidence base, as well as the small absolute effect size and borderline statistical significance. Further study is needed to better define and quantify these associations, but alcohol consumption should be considered a potential modifiable risk factor for the development of glaucoma. In particular, future research is needed to better define the dose-dependent associations of alcohol with various glaucoma-related outcomes and traits, as well as the gene–alcohol interactions underpinning these associations. Large-scale observational studies and newer genetic epidemiological techniques also offer potential avenues for further investigation, including the use of genetic proxies of alcohol consumption (Mendelian randomization),^101^ objective structural glaucoma biomarkers (including inner retinal OCT measures and cup-disc ratios), and polygenic risk scores.^102^ As the global burden of glaucoma is projected to increase further over the coming decades, ongoing investigation into environmental risk factors, as well as gene–environment interactions, is necessary to improve our understanding of glaucoma pathogenesis and potentially lead to novel preventative measures and treatment strategies.

## Supplementary Data

Appendix C

List of members of the Modifiable Risk Factors for Glaucoma Collaboration
