# Supplementary material for: Alcohol, Intraocular Pressure, and Open-Angle Glaucoma: A Systematic Review and Meta-analysis
Source: Ophthalmology. Author manuscript; Available in PMC 2022 Jun 1. (PMC9126073; doi:10.1016/j.ophtha.2022.01.023)

Figure 3. Overall (top) and stratified (bottom) funnel plots of studies included in meta-analysis of alcohol use and open-angle glaucoma

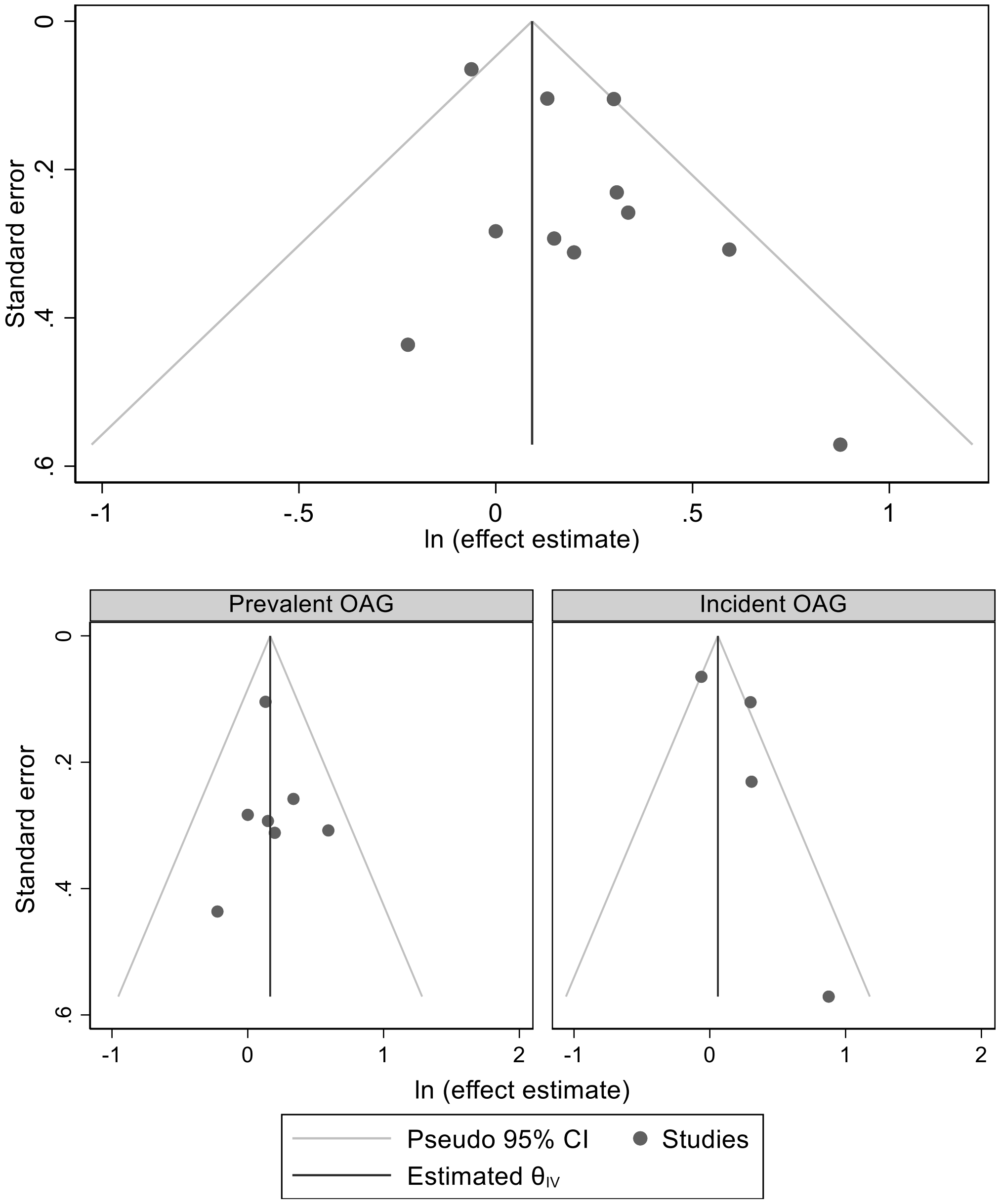

Supplement: Figure S3 [file NIHMS1788007-supplement-Figure_S3.pdf]
