# Supplementary material for: Alcohol, Intraocular Pressure, and Open-Angle Glaucoma: A Systematic Review and Meta-analysis
Source: Ophthalmology. Author manuscript; Available in PMC 2022 Jun 1. (PMC9126073; doi:10.1016/j.ophtha.2022.01.023)
Supplement: Appendix B [file NIHMS1788007-supplement-Appendix_B.pdf]

Appendix B. Funding and conflict of interest statements for all studies included in systematic review

Study (year)	Funding and conflict of interest statements
Bikbov (2020)	Open Access funding enabled and organized by Projekt DEAL. Jost B. Jonas: Advisory Board Novartis; Patent holder with Biocompatibles UK Ltd. (Farnham, Surrey, UK) (Title: Treatment of eye diseases using encapsulated cells encoding and secreting neuroprotective factor and/or anti-angiogenic factor; Patent Number: 20120263794), and Europäische Patentanmeldung 16 720 043.5 and Patent application US 2019 0085065 A1 "Agents for use in the therapeutic or prophylactic treatment of myopia or hyperopia); Songhomitra Panda-Jonas: Patent holder with Biocompatibles UK Ltd. (Farnham, Surrey, UK) (Title: Treatment of eye diseases using encapsulated cells encoding and secreting neuroprotective factor and/or anti-angiogenic factor; Patent No. 20120263794), and Europäische Patentanmeldung 16 720 043.5" Agents for use in the therapeutic or prophylactic treatment of myopia or hyperopia). All other authors: No financial disclosures.
Bonomi (2000)	Supported in part by Regione Trentino Alto Adige, Bolzano, and Merck, Sharp, and Dohme, Roma, Italy.
Charliat (1994)	None reported.
Charlson (2015)	The author(s) have made the following disclosure(s): E.S.C., P.S.S., E.M-E., M.R., J.S., R.J.S., A.L.R., B.T.T., M.C., D.W.C., H.V.G., V.R.M.C., C.N. and J.M.O. received funding from the National Eye Institute. M.P. received funding from the National Institutes of Health. G.-S.Y. received funding from the National Eye Institute and personal fees from Janssen. Supported by the National Eye Institute, Bethesda, Maryland (grant #1RO1EY023557-01) and the Department of Ophthalmology at the Perelman School of Medicine, UPenn, Philadelphia, Pennsylvania. Funds also were received from the F.M. Kirby Foundation, Research to Prevent Blindness (grant no. GFW8296), The Paul and Evanina Bell Mackall Foundation Trust, and the National Eye Institute, National Institutes of Health, Department of Health and Human Services, under eyeGENETM and contract nos. HHSN260220700001C and HHSN263201200001C. The sponsor or funding organization had no role in the design or conduct of this research.
Chiam (2018)	Conflict of interest: None. This study was funded by A-Star Biomedical Research Council (BMRC) Translational Clinical Research Program grant (TCR 0101675/BMRC Grant No: 10/1/35/19/675) and a grant (SHF/FG576S/2012) from the SingHealth Foundation in Singapore.
Doshi (2008)	Support: National Eye Institute, Bethesda, Maryland, and National Center on Minority Health and Health Disparities, Bethesda, Maryland (grant nos. EY 11753, EY 03040), and Research to Prevent Blindness, New York, New York (unrestricted grant). Dr Varma is a Research to Prevent Blindness Sybil B. Harrington Scholar. The authors have no proprietary or commercial interest in any materials discussed in the article.
Fan (2004)	This study was supported by a grant from Medicine Panel, Chinese University of Hong Kong (No. 2040820).
Jiang (2012)	The authors have no proprietary or commercial interest in any of the materials discussed in this article. Supported by the National Institutes of Health Grants NEI U10-EY-11753 and EY-03040 and an unrestricted grant from the Research to Prevent Blindness, New York, New York, and Pfizer Inc. Rohit Varma is a Research to Prevent Blindness Sybil B. Harrington Scholar. The sponsor or funding organization had no role in the design or conduct of this research.
Kaimbo (2001)	None reported.
Kang (2007)	This work was supported by grants CA87969, CA55075, EY09611, HL35464, and EY015473 from the National Institutes of Health.
Katz (1998)	This study was supported by Research Grant EY03605 from the National Eye Institute, National Institutes of Health, Bethesda, Maryland.
Klein (1993)	Supported by National Institute of Health National Eye Institute grants EY06594 and EY08012, Bethesda, Maryland.
Lee (2019)	No potential conflict of interest relevant to this article was reported.
Lee (2020)	This research received no external funding. The authors declare no conflicts of interest.
Leske (1996)	This work was supported by grant ROr EYo3684 from the National Eye Institute.
Leske (2001)	Supported by the National Eye Institute, Bethesda, Maryland (grant no.: EY01100).
Lin (2005)	Financial disclosure: None. The study was supported by grants from Taipei Veterans General Hospital (VGH 89-404 and VGH 92-136) and by Yen Tjing Ling Medical Foundation (Dr Cheng), Taipei.
Liu (2019)	The authors declare that they have no conflict of interests.
Mwanza (2018)	The author(s) have no proprietary or commercial interest in any materials discussed in this article. Supported by the Glaucoma Research Foundation, San Francisco, California; and Research to Prevent Blindness, Inc., New York, New York (unrestricted grant).
Nusinovici (2020)	Funding: NMRC/CIRG/1488/2018 and NMRC/OFLCG/004a/2018. Competing interest: None declared.
Pan (2017)	This study was supported by the National Natural Science Foundation Project of China (81460085; 81160121; 81371016). Financial disclosures: None.

Appendix B. Funding and conflict of interest statements for all studies included in systematic review (continued)

Study (year)	Funding and conflict of interest statements
Ramdas (2011)	This work was supported by Topcon Europe BV, Capelle aan de IJssel, the Netherlands, and Heidelberg Engineering, Dossenheim, Germany. This work was supported by Stichting Lijf en Leven, Krimpen aan de Lek, the Netherlands; MD Fonds, Utrecht, the Netherlands; Rotterdamse Vereniging Blindenbelangen, Rotterdam, the Netherlands; Stichting Oogfonds Nederland, Utrecht; Blindenpenning, Amsterdam, the Netherlands; Blindenhulp, the Hague, the Netherlands; Algemene Nederlandse Vereniging ter Voorkoming van Blindheid (ANVVB), Doorn, the Netherlands; Landelijke Stichting voor Blinden en Slechtzienden, Utrecht; Swart van Essen, Rotterdam; Stichting Winckel-Sweep, Utrecht; Henkes Stichting, Rotterdam; Laméris Ootech BV, Nieuwegein, the Netherlands; and Medical Workshop, de Meern, the Netherlands. The sponsors or funding organizations had no role in the design, conduct, analysis, or publication of this research.
Renard (2012)	This study was sponsored by Pfizer Inc., France.
Seddon (1983)	This study was supported by National Institutes of Health training grant in the visual sciences EY07045.
Song (2020)	This research received no external funding.
Sun (2011)	The authors declare no conflict of interest. This study was supported by Special Fund for Major Research Projects (2008-02) and PhD Research Fund (BS2010-16) of the Second Hospital of HMU, China.
Topouzis (2011)	The Thessalonika Eye Study is supported in part by the International Glaucoma Association, London, United Kingdom; The UCLA Center for Eye Epidemiology, Los Angeles, California; The Health Future Foundation, Creighton University, Omaha, Nebraska; Texas Tech University Health Sciences Center, Lubbock, Texas; Pfizer, Inc, New York, New York; Merck and Co, Inc, Whitehouse Station, New Jersey; Pharmacia Hellas, Athens, Greece. All the grants were unrestricted. Dr Topouzis is on Advisory Board for Pfizer Ophthalmics and Merck & Co; has received lecture fees from Alcon Laboratories Inc and Pfizer Inc and has received grant support from Alcon Laboratories and Pfizer, Inc. Dr Harris is on the advisory board of Pfizer, Alcon, Merck & Co, and Allergan; has received lecture fees from Pfizer, Alcon, Merck & Co, and Allergan; and has received grant support from Pfizer, Merck & Co, and Allergan. Dr Coleman is Advisory Board for Allergan and Science Based Health and received grant support from Allergan. None of the authors have any financial interest or any conflict of interest related to the subject matter.
Weih (2001)	This study was supported in part by the National Health and Medical Research Council, Canberra, Australia; and the Victorian Health Promotion Foundation, the Estate of Dorothy Edols, the Ansell Ophthalmology Foundation, and the Jack Brockhoff Foundation, Melbourne, Australia.
Wise (2011)	Supported by the National Cancer Institute at the National Institutes of Health [CA058420].
Wu (1997)	Supported by grants 07625 and EY07617 from the National Eye Institute, Bethesda.
Xu (2009)	The author(s) have no proprietary or commercial interest in any materials discussed in this article. Funding: Beijing Key Laboratory Funding, Beijing, China.
Yavaş (2012)	This study was supported by Afyon Kocatepe University, Scientific Research Project Department, Turkey.
Yoshida (2003)	This study was supported by an official fund from the Kyorin University School of Medicine.
Zangwill (2018)	The author(s) have made the following disclosure(s): L.M.Z.: Financial support - Carl Zeiss Meditec, Heidelberg Engineering, Optovue, Inc., Topcon Medical System Inc. J.M.L.: Consultant - Alcon, Allergan, Bausch & Lomb, Carl Zeiss Meditec, Heidelberg Engineering, Reichert, Valeant Pharmaceuticals; Financial support - Bausch & Lomb, Carl Zeiss Meditec, Heidelberg Engineering, National Eye Institute, Optovue, Reichert, Topcon. C.A.G.: Financial support - Carl Zeiss Meditec, Heidelberg Engineering, SOLX. F.A.M.: Consultant - Allergan, Carl Zeiss Meditec, Novartis; Financial support - Alcon, Allergan, Bausch & Lomb, Carl Zeiss Meditec, Heidelberg Engineering, Merck, Reichert, Sensimed, Topcon. R.N.W.: Consultant - Aeries Pharmaceutical, Alcon, Allergan, Bausch & Lomb, Eyenovia, Sensimed; Financial support - Heidelberg Engineering, Carl Zeiss Meditec, Genentech, Optovue, Topcon. Supported by the National Eye Institute, National Institutes of Health, Bethesda, Maryland (grant nos.: EY023704, P30EY022589, EY110008, EY019869, and EY021818); and the National Institutes of Health (grant nos.: R01 DK087914, R01 DK066358, R01 DK053591, U01 DK105556, R01 HL56266, R01 DK070941, DRC DK063491, and CTSI UL1TR001881); the EyeSight Foundation of Alabama (C.A.G.); and Research to Prevent Blindness, Inc., New York, New York (C.A.G.).
