# Supplementary material for: Alcohol, Intraocular Pressure, and Open-Angle Glaucoma: A Systematic Review and Meta-analysis
Source: Ophthalmology. 2022 Jun;129(6):637–52. doi: 10.1016/j.ophtha.2022.01.023 (PMC9126073; doi:10.1016/j.ophtha.2022.01.023)

Figure 3. Overall (top) and stratified (bottom) funnel plots of studies included in meta-analysis of alcohol use and open-angle glaucoma

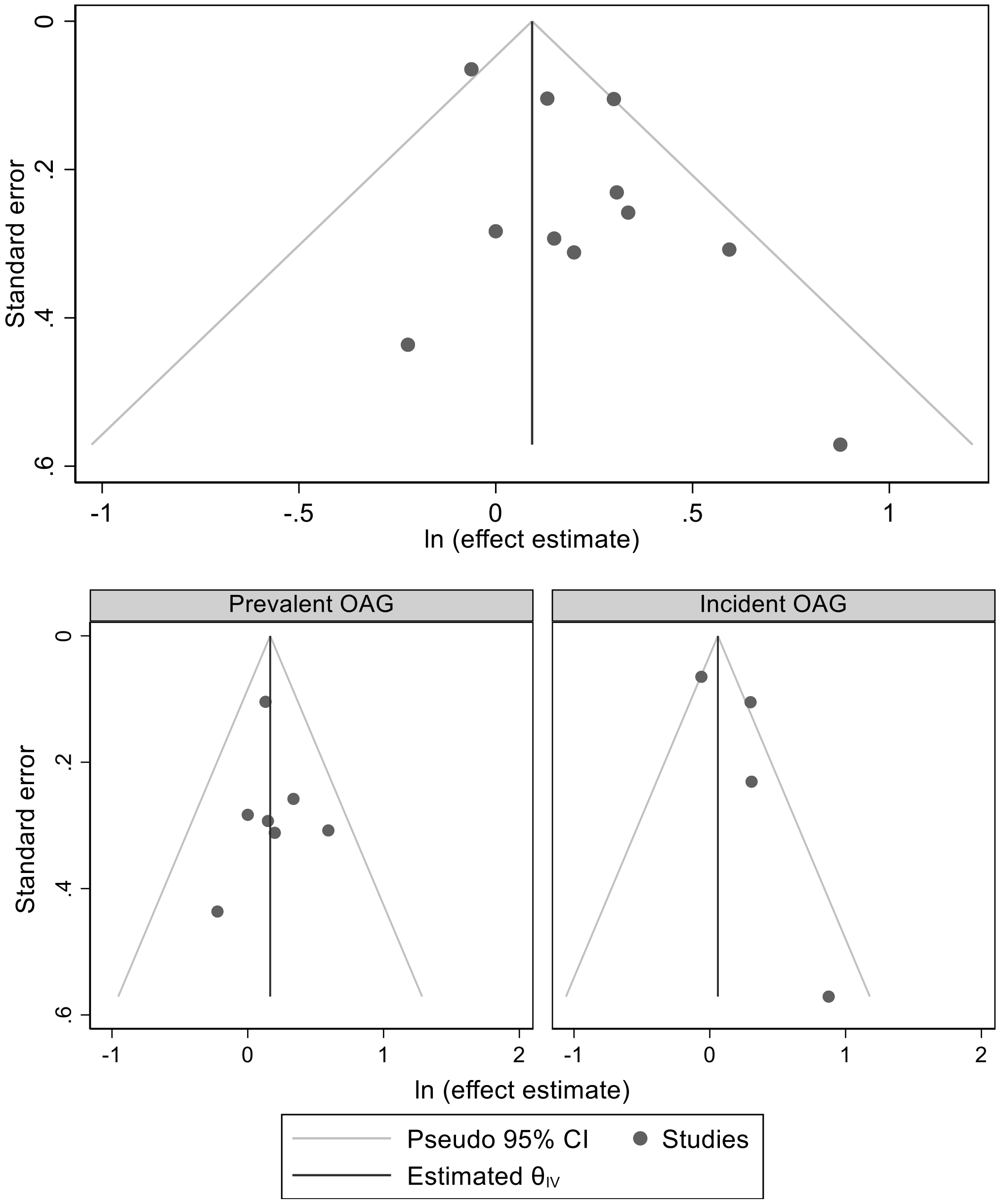

Supplement: Supplementary file 1 [file mmc1.pdf]
