# Supplementary material for: Alcohol, Intraocular Pressure, and Open-Angle Glaucoma: A Systematic Review and Meta-analysis
Source: Ophthalmology. 2022 Jun;129(6):637–52. doi: 10.1016/j.ophtha.2022.01.023 (PMC9126073; doi:10.1016/j.ophtha.2022.01.023)
Supplement: Supplementary file 2 [file mmc2.pdf]

Figure 4. Risk of bias assessment of studies included in the meta-analysis of alcohol use and open-angle glaucoma

**Study**	**Risk of bias assessment**							**Study-level assessment**
	Confounding	Participant selection	Exposure classification	Exposure departures	Missing data	Outcome measurement	Reported results	
Bikbov (2020)	Serious	Moderate	Serious	Serious	Low	Moderate	Moderate	Serious
Bonomi (2000)	Critical	Moderate	Serious	Serious	Low	Moderate	Moderate	Critical
Charliat (1994)	Serious	Serious	Serious	Serious	Low	Low	Moderate	Serious
Chiam (2018)	Serious	Moderate	Moderate	Moderate	Low	Low	Moderate	Serious
Leske (1996)	Serious	Serious	Serious	Serious	Low	Moderate	Moderate	Serious
Leske (2001)	Critical	Serious	Serious	Serious	Low	Low	Moderate	Critical
Renard (2013)	Serious	Serious	Moderate	Moderate	Low	Serious	Moderate	Serious
Jiang (2012)	Serious	Moderate	Moderate	Moderate	Moderate	Low	Moderate	Serious
Kang (2007)	Moderate	Moderate	Low	Moderate	Moderate	Moderate	Moderate	Moderate
Pan (2017)	Serious	Moderate	Serious	Serious	Moderate	Low	Moderate	Serious
Wise (2011)	Moderate	Moderate	Moderate	Serious	Moderate	Moderate	Moderate	Serious
**Domain-specific assessment**	Critical	Serious	Serious	Serious	Moderate	Serious	Moderate	

Risk of bias key:

Low
Moderate
Serious
Critical
