# Supplementary material for: Alcohol, Intraocular Pressure, and Open-Angle Glaucoma: A Systematic Review and Meta-analysis
Source: Ophthalmology. 2022 Jun;129(6):637–52. doi: 10.1016/j.ophtha.2022.01.023 (PMC9126073; doi:10.1016/j.ophtha.2022.01.023)
Supplement: Supplementary file 3 [file mmc3.pdf]

Table 2. Case definition of open-angle glaucoma used by studies included in systematic review

**Author (year)**	**Direct examination**	**Clinical review / Record linkage**	**VF assessment**	**ONH assessment**	**Angle assessment**	**Exclude / adjust for other OAG**	**Independent of IOP**
Bikbov (2020)	✓	-	✓	✓	✓[1]	N/A	✓[2]
Bonomi (2000)	✓	-	✓	✓	✓	-	-
Charliat (1994)	✓	-	✓	✓	✓[1]	✓	✓
Charlson (2015)	✓	-	✓	✓	✓	✓	✓
Chiam (2018)	✓	-	✓	✓	✓	✓	✓
Fan (2004)	✓	-	✓	✓	✓[1]	✓	✓
Jiang (2012)	✓	-	✓	✓	✓	N/A	✓
Kaimbo (2001)	✓	-	✓	✓	✓	✓	-
Kang (2007)	-	✓	✓	-	✓	✓	✓
Katz (1988)	✓	Supplementary	✓	-	-	-	-
Klein (1993)	✓	-	✓	✓	-	N/A	-
Lee (2020)	✓	-	✓	✓	✓[1]	✓	✓
Leske (1996)	-	✓	✓	✓	-	N/A	-
Leske (2001)	✓	Supplementary	✓	✓	✓	N/A	✓
Liu (2020)	✓	-	✓	✓	✓	✓	✓
Mwanza (2019)	✓	-	✓	✓	✓	N/A	✓[2]
Nusinovici (2020)	✓	-	✓	✓	✓	✓	✓[2]
Pan (2017)	✓	-	✓	✓	✓	✓	✓[2]
Ramdas (2011)	✓	-	✓	✓	✓[1]	N/A	✓
Renard (2013)	✓	-	✓	✓	✓[1]	✓	-
Sun (2012)	✓	-	✓	✓	✓	✓	✓[2]
Topouzis (2011)	✓	-	✓	✓	✓	✓	-
Wise (2011)	-	✓	N/A	N/A	N/A	✓	N/A
Xu (2009)	✓	-	-	✓	✓[1]	N/A	✓
Yavaş (2013)	✓	-	✓	✓	✓	-	-
Zangwill (2019)	-	✓	✓	✓	✓	✓	✓

[1] Method of angle assessment not gonioscopy (e.g., van Herick technique, anterior segment OCT) or not reported.

[2] Case ascertainment based on the International Society of Geographical and Epidemiologic Ophthalmology (ISGEO) criteria in which category III includes IOP >99.5th percentile as a component of the definition of glaucoma.

VF, visual field; ONH, optic nerve head; OAG, open-angle glaucoma; IOP, intraocular pressure; OCT, optical coherence tomography.
