# Supplementary material for: Alcohol, Intraocular Pressure, and Open-Angle Glaucoma: A Systematic Review and Meta-analysis
Source: Ophthalmology. 2022 Jun;129(6):637–52. doi: 10.1016/j.ophtha.2022.01.023 (PMC9126073; doi:10.1016/j.ophtha.2022.01.023)
Supplement: Supplementary file 4 [file mmc4.pdf]

Table 3. Characteristics of studies reporting an association between alcohol use and open-angle glaucoma but not meeting criteria for inclusion in meta-analysis

Author (year)	Location (study)	Design	Population	Size (cases)	Result & effect estimate (95% CI)	Exclusion
Charlson (2015)	USA (Primary Open-Angle African American Glaucoma Genetics Study	CC	≥40 years	2067 (807)	History of alcohol use protective for prevalent POAG, OR 0.77 (0.63-0.93). No association after adjustment for age (data not reported).	1
Fan (2004)	China	CC	35-83 years	128 (96)	Alcohol consumption (average ≥100mls liquor daily for ≥1 year) protective for prevalent POAG, OR 0.03 (0.00-0.55).	2
Kaimbo (2001)	Democratic Republic of Congo	CC	28-80 years	144 (104)	Alcohol consumption not associated with prevalent OAG, OR 0.96 (0.39-2.40).	1
Katz (1998)	USA (Nerve Fiber Layer Study)	CC	Mean 59 years	188 (94)	Alcohol consumption (>28 grams daily) not associated with prevalent POAG, OR 1.46 (0.72-2.96). Harmful association in white participants (OR 2.43, 1.01-5.86) but not black participants.	2
Klein (1993)	USA (Beaver Dam Eye Study)	CS	43-84 years	4926 (104)	Current alcohol use not associated with prevalent OAG in men (OR 1.41, 0.54-3.64) or women (OR 0.59, 0.33-1.03). No association with a history of heavy drinking. No interaction with cigarette smoking.	1
Lee (2020)	South Korea (Korea National Health and Nutrition Examination Survey)	CS	≥20 years	6742 (323)	Alcohol drinking (≥1 drinks/week) not associated with prevalent POAG, OR 1.01 (0.80-1.27).	1, 2
Liu (2020)	China	CC	Mean 64 years	973 (650)	Alcohol drinking harmful for prevalent POAG, OR 1.38 (1.04-1.83) on univariable analysis. Evidence of gene-alcohol interaction (see *Discussion*) on multivariable analysis.	1, 2
Mwanza (2019)	Ghana (Tema Eye Survey)	C	≥40 years	1101 (51)	Alcoholism (regular consumption of any amount of alcohol) not associated with incident OAG, OR 1.01 (0.95-1.09).	2
Nusinovici (2020)	Singapore (Singapore Epidemiology of Eye Disease Study)	CS	40-80 years	5027 (209)	Alcohol consumption: 1/week (OR 0.86, 0.33-1.83) and ≥2/week (OR 0.77, 0.34-1.51) not associated with prevalent POAG.	1
Ramdas (2011)	Netherlands (Rotterdam Study)	C	≥55 years	3939 (108)	Any alcohol intake not associated with incident OAG, OR 1.15 (0.64-2.21). No association with grams/day or alcohol type.	1
Sun (2012)	China	CS	≥40 years	4956 (35)	Alcohol consumption not associated with prevalent POAG, OR 0.89 (0.66-1.20).	1
Topouzis (2011)	Greece (Thessalonika Eye Study)	CS	≥60 years	1991 (94)	Regular alcohol consumption (≥1 drinks/week) not associated with prevalent POAG, OR 0.99 (0.64-1.55).	1, 2
Xu (2009)	China (Beijing Eye Study)	CS	≥40 years	4141 (21)	Alcohol consumption not associated with prevalent OAG, OR 1.09 (0.21-3.76).	1
Yavaş (2013)	Turkey	CS	≥40 years	1533 (30)	Alcohol consumption not associated with prevalent POAG, OR 2.33 (0.58-6.93).	1
Zangwill (2019)	USA (African Descent and Glaucoma Evaluation Study)	CC	Mean 67 years	3267 (425)	Current alcohol consumption protective for prevalent POAG in black participants (OR 0.78, 0.63-0.97) but harmful in white participants (OR 1.67, 1.22-2.29). Significant associations after adjustment for age (data not reported).	1

Reason for exclusion from meta-analysis: (1) no multivariable effect estimate, (2) reference group included drinkers or excluded non-drinkers.
OAG, open-angle glaucoma; POAG, primary open-angle glaucoma; CS, cross-sectional; CC, case-control; C, cohort; OR, odds ratio; CI, confidence interval.
