# Supplementary material for: Alcohol, Intraocular Pressure, and Open-Angle Glaucoma: A Systematic Review and Meta-analysis
Source: Ophthalmology. 2022 Jun;129(6):637–52. doi: 10.1016/j.ophtha.2022.01.023 (PMC9126073; doi:10.1016/j.ophtha.2022.01.023)
Supplement: Supplementary file 5 [file mmc5.pdf]

Appendix A. Search strategy

Database: ***PubMed*** **(1231 results)**

("alcohol*" OR "alcohol drinking" [MeSH] OR "ethanol" OR "beer" OR "wine" OR "spirit*" OR "diet*" OR "life?style") AND ("glaucoma*" OR "ocular hypertension" [MeSH] OR "intra?ocular pressure" OR "?OAG" OR "IOP") [Limits: English]

Database: ***Embase*** **(2338 results)**

1	alcohol*.mp. (669861)
2	ethanol.mp. (159686)
3	beer/ or wine/ or spirit*/ (19598)
4	diet*.mp. (999474)
5	life?style.mp. (226108)
6	1 or 2 or 3 or 4 or 5 (1809083)
7	glaucoma*.mp. (91406)
8	ocular hypertension.mp. (6851)
9	intra?ocular pressure.mp. (66369)
10	(POAG or OAG).mp. (7468)
11	IOP.mp. (35767)
12	7 or 8 or 9 or 10 or 11 (131940)
13	6 and 12 (2470)
14	Limit 13 to English language (2338)

Database: ***Scopus*** **(1632 results)**

TITLE-ABS-KEY (("alcohol*" OR "ethanol" OR "beer" OR "wine" OR "spirit*" OR "diet*" OR "life?style") AND ("glaucoma*" OR "ocular hypertension" OR "intra?ocular pressure" OR "?OAG")) AND (LIMIT-TO (LANGUAGE,"English"))
