# Supplementary material for: Alcohol, Intraocular Pressure, and Open-Angle Glaucoma: A Systematic Review and Meta-analysis
Source: Ophthalmology. 2022 Jun;129(6):637–52. doi: 10.1016/j.ophtha.2022.01.023 (PMC9126073; doi:10.1016/j.ophtha.2022.01.023)
Supplement: Supplementary file 8 [file mmc8.pdf]

List of members of the Modifiable Risk Factors for Glaucoma Collaboration

Hugues Aschard, Institute Pasteur, Paris, France

Mark Chia, Moorfields Eye Hospital NHS Foundation Trust & UCL Institute of Ophthalmology, London, UK

Sharon Y.L. Chua, Moorfields Eye Hospital NHS Foundation Trust & UCL Institute of Ophthalmology, London, UK

Ron Do, Icahn School of Medicine at Mount Sinai, New York, NY, USA

Pirro G. Hysi, King's College London, St. Thomas' Hospital, London, UK

Paul J. Foster, Moorfields Eye Hospital NHS Foundation Trust & UCL Institute of Ophthalmology, London, UK

Jae H. Kang, Brigham and Women's Hospital, Harvard Medical School, Boston, MA, USA

Alan Kastner, Moorfields Eye Hospital NHS Foundation Trust, London, UK

Anthony P. Khawaja, Moorfields Eye Hospital NHS Foundation Trust & UCL Institute of Ophthalmology, London, UK

Jihye Kim, Harvard T.H. Chan School of Public Health, Boston, MA, USA

Marleen AH Lentjes, Örebro University, Campus USÖ, Örebro, Sweden

Robert N. Luben, University of Cambridge School of Clinical Medicine, Cambridge, UK

Kian Madjedi, University of Calgary, Alberta, Canada

Giovanni Montesano, Moorfields Eye Hospital NHS Foundation Trust, London, UK

Louis R. Pasquale, Icahn School of Medicine at Mount Sinai, New York, NY, USA

Kelsey V. Stuart, Moorfields Eye Hospital NHS Foundation Trust & UCL Institute of Ophthalmology, London, UK

Jessica H. Tran, Icahn School of Medicine at Mount Sinai, New York, NY, USA

Alasdair N. Warwick, UCL Institute of Cardiovascular Science, London, UK

Janey L. Wiggs, Massachusetts Eye and Ear Infirmary, Harvard Medical School, Boston, MA, USA
